# Reviewed article: Lima GCC, Passos CM, Pinheiro ALS, Ribeiro IJS, Maia EG. Temporal trend and epidemiological profile of notifications of violence against women in Brazil: 2014 to 2023. Epidemiol Serv Saude. 2025:34;e20240475.

**DOI:** 10.1590/S2237-96222025v34e20240475.b

**Published:** 2025-08-08

**Authors:** Christine Faustino

**Affiliations:** Afiliação Avaliador UEMS, Medicine

**Table:** 

Rating	Excellent	Good	Average	Below average	Poor
Interest			✓		
Quality			✓		
Originality		✓			
Overall			✓		

**Table:** 

Questionnaire	Yes	No	Not applicable
Does the manuscript contain new and significant information to justify publication?		✓	
Does the Abstract (Summary) clearly and accurately describe the content of the article?	✓		
Is the problem significant and concisely stated?	✓		
Are the methods described comprehensively?	✓		
Are the interpretations and conclusions justified by the results?	✓		
Is adequate reference made to other work in the field?		✓	

## Manuscript Structure

Length of article is: Adequate

Number of tables is: Adequate

Number of figures is: Adequate

Please state any conflict(s) of interest that you have in relation to the review of this paper (state “none” if this is not applicable): None

## Comments

O tema escolhido é bastante relevante, porém recomenda-se a revisão maior, pois o SINAN disponibiliza dados até 2023. Deste modo, orienta-se a inclusão da análise dos dados de 2020 até 2023, porque o recorte escolhido não apresenta interpretação atualizada sobre o assunto. A versão do texto passa a impressão de um trabalho obsoleto na coleta dos dados e na redação. O estudo é uma análise de série temporal erroneamente denominado de estudo ecológico. Outros itens que necessitam ser revisados: a hipótese inicial não foi declarada, as referências não incluem os trabalhos mais recentes, o método de escolha das categorias das variáveis não foi definido, as inconsistências internas encontradas no banco de dados e os tratamentos empregados não foram descritos e as possíveis contribuições ao Sistema Único de Saúde não foram mencionadas.

